# Characterization of Vesicle Differentiation Mutants of *Frankia casuarinae*

**DOI:** 10.1264/jsme2.ME19150

**Published:** 2020-04-07

**Authors:** Koya Asukai, Ken-ichi Kucho

**Affiliations:** 1 Graduate School of Science and Engineering, Kagoshima University, 1–21–35 Korimoto, Kagoshima 890–0065, Japan

**Keywords:** glutamine synthetase, multicellular bacteria, nitrogen fixation, vesicle

## Abstract

The nitrogen-fixing actinobacterium *Frankia* develops unique multicellular structures called vesicles, which are the site of nitrogen fixation. These vesicles are surrounded by a thick hopanoid lipid envelope that protects nitrogenase against oxygen inactivation. The phenotypes of five mutants that form smaller numbers of vesicles were investigated. The vesicles of these mutants were smaller than those of the wild type and had a phase dark appearance. They induced the expression of a glutamine synthetase gene in hyphae cells in response to ammonium starvation. These results suggest that genes impaired in the mutants do not function in global nitrogen regulation, but specifically function in vesicle differentiation.

Nitrogen is an essential element for all living organisms. Most organisms cannot utilize dinitrogen gas (N_2_) because of its stable triple bound. Nitrogen-fixing (N_2_-fixing) bacteria have the ability to reduce N_2_ to ammonia (NH_3_), and assimilate it to organic compounds such as amino acids. Fixed nitrogen flows into ecology, and, thus, N₂-fixing bacteria play an important role in global nitrogen cycles.

N_2_ fixation is catalyzed by nitrogenase, which is a complex metalloenzyme composed of dinitrogenase (NifDK) and dinitrogenase reductase (NifH) (Dixon and Kahn, 2004). Since nitrogenase is highly oxygen-labile, N_2_-fixing bacteria adopt diverse strategies (behavioral, physiological, and structural) to protect nitrogenase against oxygen inactivation (Gallon, 1992).

*Frankia* spp. are N_2_-fixing multicellular actinobacteria. Under NH_3_-depleted and aerobic conditions, *Frankia* develop spherical multicellular structures called vesicles (Fig. S1), which are the site for N_2_ fixation (Huss-danell, 1997). These vesicles are surrounded by a thick envelope composed of dozens of hopanoid lipid layers (Berry *et al.*, 1993). Since the envelope functions as a barrier to oxygen penetration, nitrogenase, which is expressed inside vesicles, retains its activity (Benson and Silvester, 1993). Vesicles are not formed under anaerobic conditions and N_2_ fixation occurs in hyphal cells (Murry *et al.*, 1985). Genes related to vesicle differentiation have not yet been identified, except for those related to hopanoid lipid synthesis, which are ubiquitous in the microbial world (Kannenberg and Poralla, 1999).

We previously isolated five N_2_-fixation mutants of *Frankia casuarinae* (G21E10, G23C4, G23D3, N7C9, and N10E6), which had smaller numbers of vesicles (<*ca.* 15% of the wild type) (Kucho *et al.*, 2017) (Table S1). These mutants are considered to have defects in the generation of vesicle primordia. In the present study, we characterized the phenotypes of these mutants in more detail.

We used *F. casuarinae* strain CcI3 as the wild type (WT) (Nouioui *et al.*, 2016). *Frankia* strains were grown in NH_3_-repleted (N+) BAP-TN+ liquid medium (Kucho *et al.*, 2009) at 28°C with stirring until the mid-logarithmic phase, and cells were then transferred to NH_3_-depleted (N–) BAP-TN– medium (Kucho *et al.*, 2009). Vesicles were observed 7‍ ‍d after being transferred to N– conditions using phase-contrast (for size measurements) and dark-field (for envelope evaluations) optical systems with the microscope MT5310L (Meiji Techno). The vesicle sizes of G21E10, G23D3, N7C9, and N10E6 were markedly smaller than those of WT (<60%), while those of G23C4 were slightly smaller (80% of WT) (Fig. 1 and Table S1). When observed under the dark-field microscope, the thickness of the envelope was proportional to its brightness (Parsons *et al.*, 1987) because the light effect was attributed to birefringence produced by structural layering of the vesicle envelope. In WT, approximately 40% of vesicles showed a bright appearance, indicative of a well-developed envelope (Fig. 2 asterisk and Fig. S2). Approximately 60% of WT vesicles also had a thick-walled stalk (Fig. 2 arrowhead and Fig. S2). In all mutants, the frequency of vesicles with a well-developed envelope was significantly less than that in WT and was markedly lower in G23C4 (5%) and N10E6 (0%) (Fig. 2 and S2). Furthermore, G23D3 and N10E6 produced fewer vesicles with a thick-walled stalk (Fig. 2 and S2). These results indicate that genes impaired in these mutants are important not only for the generation of primordia, but also for the maturation of vesicles (size expansion and envelope development).

The genes impaired in these mutants may be directly involved in the vesicle differentiation process. Alternatively, these genes may function in the perception or signaling of a NH_3_-starvation status and their mutations indirectly disabled downstream vesicle differentiation (Fig. 3). To clarify the site of function, we investigated the expression of a NH_3_-responsive gene in hyphal cells. If the latter is the case, these mutants will not be able to induce gene expression in hyphal cells or induce vesicle formation (Fig. 3). Therefore, we focused on a glutamine synthetase (GS) gene, which converts NH_3_ and glutamate to glutamine. *Frankia* has two types of GS enzyme—GSI and GSII—that show distinct biochemical and regulatory properties, and the expression of the GSII gene was previously shown to be up-regulated in hyphal cells (and in vesicles) in response to NH_3_-starvation (Schultz and Benson, 1990; Ghodhbane-Gtari *et al.*, 2014). We also investigated the expression of a gene involved in NH_3_-responsive regulation (*ntrB*, see below).

*Frankia* cells were acclimated to N– conditions as described above. Cells were collected by centrifugation 4‍ ‍d after being transferred to N– conditions, and total RNA was purified by the cetyltrimethylammonium bromide (CTAB) method (Kucho *et al.*, 2009). Contaminating DNA was removed by the TURBO DNA-free kit (Thermo Fisher Scientific). The cDNAs of the GSII (*francci3_3143*), *ntrB* (*francci3_3178*), and 16S rRNA (*francci3_R0040*, internal standard) genes were synthesized using PrimeScript reverse transcriptase (Takara Bio) in a 20-μL reaction mixture containing 1.5‍ ‍μg of total RNA and 2 pmol of gene-specific reverse primers (GSII, Ghodhbane-Gtari *et al.*, 2014; 16S rRNA, Kucho *et al.*, 2017; *ntrB*, 5′-cccacatctcgggcagtt-3′) at 42°C for 30‍ ‍min and then at 50°C for 15‍ ‍min. Regarding GSII and 16S rRNA, real-time PCR was performed using the Probe qPCR mix (Takara Bio) in a 20-μL reaction mixture containing 4 pmol of forward and reverse primers (GSII, Ghodhbane-Gtari *et al.*, 2014; 16S rRNA, Kucho *et al.*, 2017), 4 pmol of the TaqMan probe (GSII, 5′-acgccatcgtcgcctgct-3′; 16S rRNA, Kucho *et al.*, 2017), and cDNA derived from 100‍ ‍ng (GSII) or 1‍ ‍ng (16S rRNA) of total RNA. Regarding *ntrB* and 16S rRNA, semi-quantitative PCR was performed using *EX Taq* DNA polymerase (Takara Bio) in a 20-μL reaction mixture containing 4 pmol of a forward primer (*ntrB*, 5′-gccgctgaccagtgtgaa-3′; 16S rRNA, Kucho *et al.*, 2017) and reverse primer (same primers used in reverse transcription), and cDNA derived from 100‍ ‍ng (*ntrB*) or 1‍ ‍ng (16S rRNA) of total RNA. A temperature regime (95°C for 30‍ ‍s, 58°C for 30‍ ‍s, and 72°C for 18 s) was repeated 28 times for *ntrB* or 23 times for 16S rRNA.

In WT, the mRNA levels of the GSII gene were markedly higher under N– than N+ conditions (Fig. 4). Under N– conditions, all mutant strains (G21E10, G23C4, G23D3, N7C9, and N10E6) showed similar GSII expression levels to WT and these levels appeared to be higher than those of N+ WT (Fig. 4 and Table S1). These results indicate that the mutants retained the abilities to perceive NH₃ starvation and transduce the signal to the GSII gene in hyphal cells in order to activate its expression. In many eubacteria, NH_3_-responsive regulation is accomplished by the nitrogen regulation (*ntr*) system (Merrick and Edwards, 1995), and homologs of its components (*glnBD* and *ntrBC*) have been found in *Frankia* genomes. Semi-quantitative reverse transcription PCR showed that an *ntrB* homolog (*francci3_3178*) was expressed in WT and all the mutant strains (Fig. 5 and Table S1). Genome analyses revealed that three of the mutants (G23C4, N7C9, and N10E6) did not carry mutations in these homologs (Kucho *et al.*, 2017). Collectively, these results indicate that the genes responsible for the phenotypes of the three mutants are not related to global nitrogen regulation, but specifically function in the vesicle differentiation process (Fig. 3). The mutants G21E10 (Kucho *et al.*, 2017) and G23D3 (K. Kucho, unpublished) carried an identical amino acid substitution in the homolog of *ntrB* (*francci3_3178*), whereas the same mutation was found in a revertant strain that formed vesicles and fixed N_2_ (K. Kucho, unpublished). Therefore, the mutation in the *ntrB* homolog did not appear to cause the mutant phenotypes and G21E10 and G23D3 may be impaired in other genes that specifically function in the vesicle differentiation process.

Vesicle differentiation-specific genes are considered to be unique for *Frankia* and have not yet been identified. Two laboratories recently reported the successful transformation of *Frankia* spp. (Gifford *et al.*, 2019; Pesce *et al.*, 2019). Using these methods, we will be able to identify the genes responsible for the mutant phenotypes using complementation experiments with a genomic library of the WT strain.

## Citation

Asukai, K., and Kucho, K. (2020) Characterization of Vesicle Differentiation Mutants of *Frankia casuarinae*. *Microbes Environ ***35**: ME19150.

https://doi.org/10.1264/jsme2.ME19150

## Supplementary Material

Supplementary Material

## Figures and Tables

**Fig. 1.**
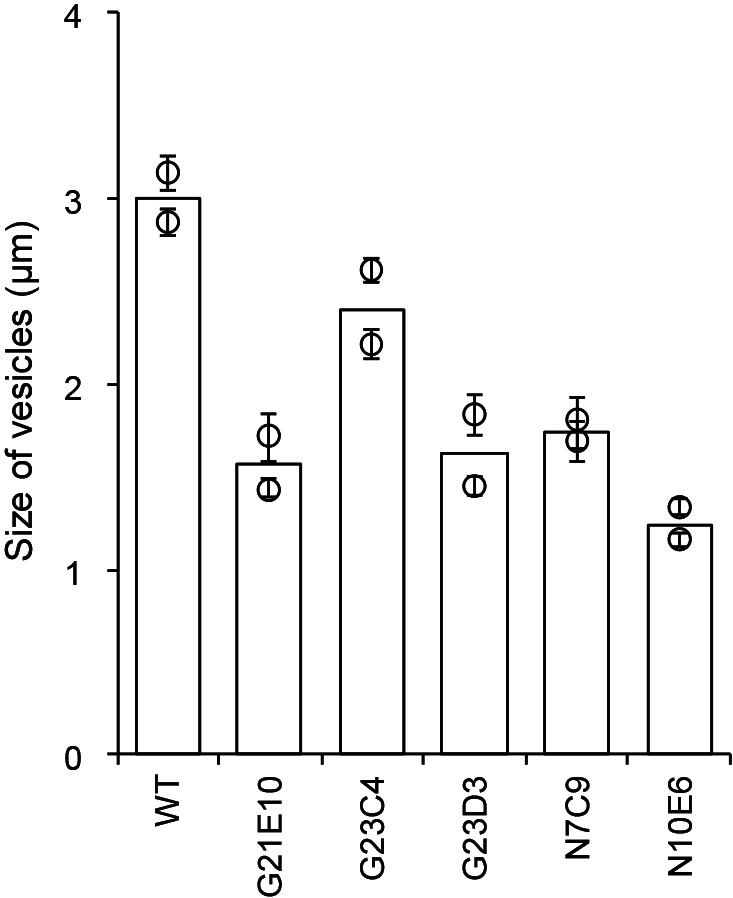
Size of vesicles. Each dot represents an average calculated from between 20 and 36 independent vesicles from a biological replicate. The bar represents the standard error. Medians calculated from two biological replicates are shown by open boxes.

**Fig. 2.**
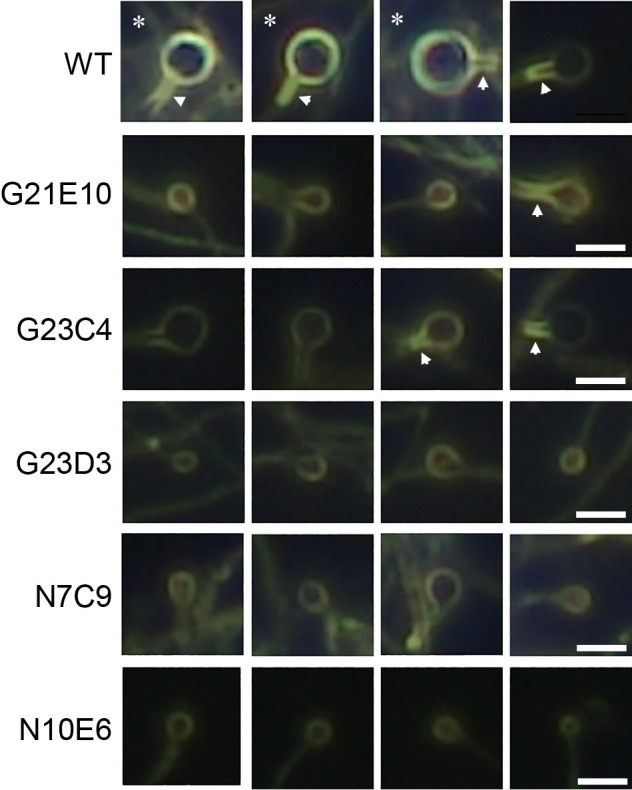
Dark-field microscopic images of vesicles. A vesicle and stalk with a well-developed envelope are shown by an asterisk and arrowhead, respectively. The bar represents 3 μm.

**Fig. 3.**
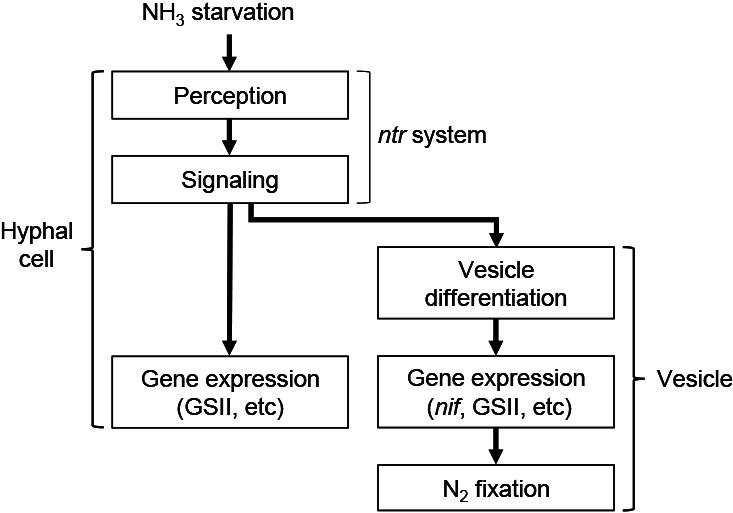
Schematic diagram of sequential events predicted to occur in *Frankia* in response to NH_3_ starvation under aerobic conditions. This is a working hypothesis that requires further evidence.

**Fig. 4.**
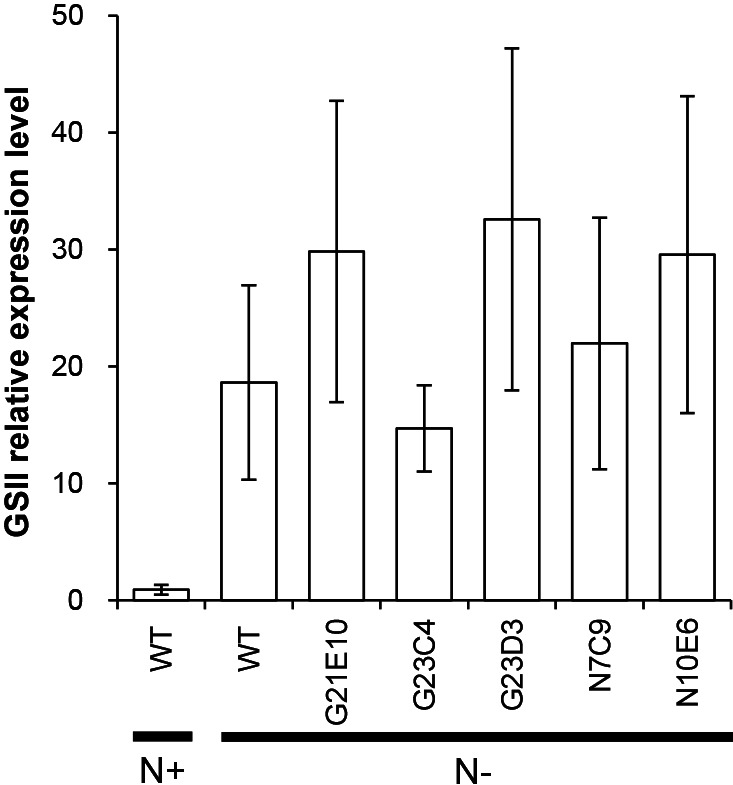
Expression of the GSII gene. Relative transcript levels to a value obtained from a WT N+ sample are shown. Averages calculated from three biological replicates are shown. The bar represents the standard error. N+, NH_3_-repleted; N–, NH_3_-depleted conditions.

**Fig. 5.**
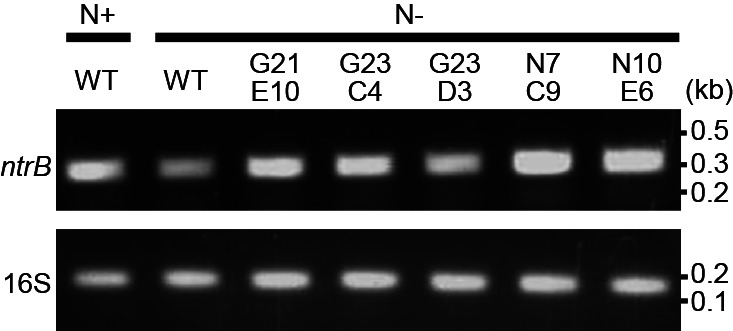
Semi-quantitative reverse transcription PCR of *ntrB* (*francci3_3178*) and 16S rRNA (16S) genes. The expected sizes of the amplified products are 299 bp (*ntrB*) and 120 bp (16S rRNA). N+, NH_3_-repleted; N–, NH_3_-depleted conditions. Signals were weak when RNA samples without reverse transcription were used as templates (data not shown).
