# Supplementary material for: Characterization of Vesicle Differentiation Mutants of Frankia casuarinae
Source: Microbes Environ. 2020 Apr 7;35(2):ME19150. doi: 10.1264/jsme2.ME19150 (PMC7308572; doi:10.1264/jsme2.ME19150)
Supplement: Supplementary file 1 — Supplementary Material [file 35_19150_s1.pdf]

Table S1. Phenotypes of the mutants used in this study.

		G21E10	G23C4	G23D3	N7C9	N10E6
Vesicle	Number [a]	13% [c]	13% [c]	4% [c]	8% [c]	17% [c]
	Size [b]	52% [c]	80% [c]	54% [c]	58% [c]	41% [c]
	Envelope[b]	19% [d]	5% [d]	23% [d]	12% [d]	0% [d]
Gene expression	*nifEHV* [a]	- [e]	-	-	-	-
	GSII [b]	+ [e]	+	+	+	+
	ntrB [b]	+	+	+	+	+
N_2-fixation-related genes carrying mutations [f]		Francci3_3178, *ntrB* homolog.	Francci3_1072, hydrogenase maturation protein HypF2.	Francci3_3178, *ntrB* homolog.	Francci3_1072, hydrogenase maturation protein HypF2.<br><br>Francci3_1942, uptake hydrogenase large subunit HupL1.<br><br>Francci3_4487, nitrogenase molybdenum-iron protein alpha chain NifD<br><br>Francci3_4488, nitrogenase iron protein subunit NifH	Francci3_0821, putative squalene/phytoene dehydrogenase.<br><br>Francci3_1072, hydrogenase maturation protein HypF2.

[a] Kucho *et al.*, 2017.

[b] This study.

[c] Relative value to the wild type *Frankia casuarinae* strain CcI3.

[d] Frequency of vesicles with well-developed envelope (WT = 42%) (Fig. S2).

[e] -, severely reduced compared to WT; +, comparable level to WT.

[f] G21E10, G23C4, N7C9 and N10E6 (Kucho *et al.*, 2017). G23D3 (K. Kucho, unpublished).

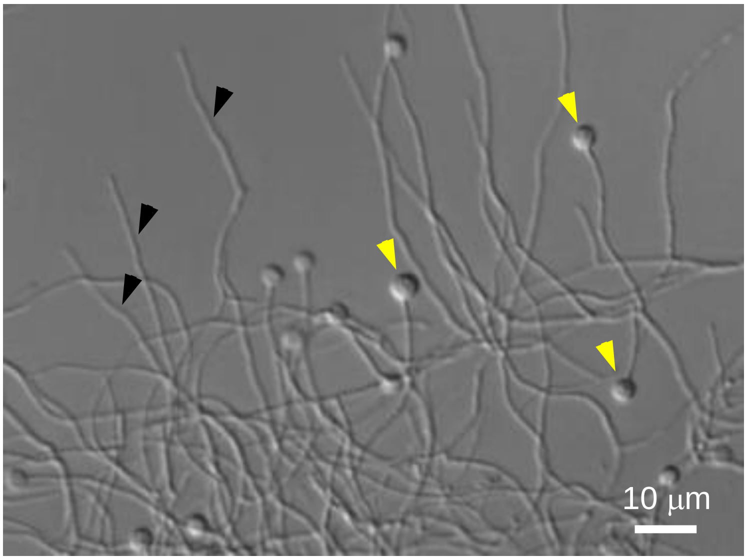

Fig. S1. Wild type *Frankia casuarinae* strain CcI3 (differential interference contrast microscope). Yellow arrowheads indicate vesicles and black arrowheads indicate hyphae.

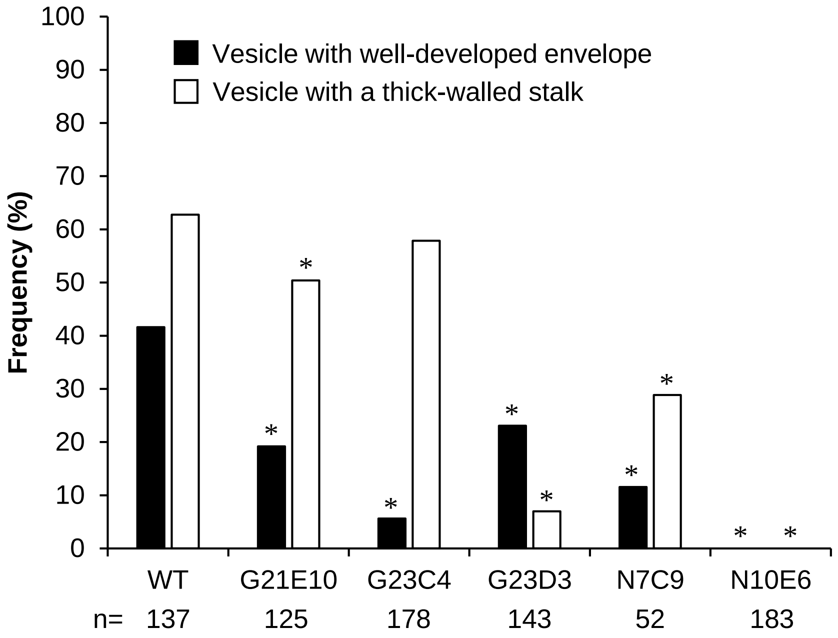

Fig. S2. Frequency of vesicles with well-developed envelope (black box) and vesicles with a thick-walled stalk (white box). Asterisk indicates significant difference with WT ($P < 0.01$ by the Chi-square test).
